# Comparative effectiveness of two behavioral change intervention packages for tobacco cessation initiated in the tertiary care setting of North India—protocol for a two-arm randomized controlled trial

**DOI:** 10.1186/s13063-022-06673-3

**Published:** 2022-09-05

**Authors:** Priyanka Dhawan, Sonu Goel, Ashutosh Aggarwal, Abhishek Ghosh, Rajesh Vijayvergiya, Bikash Medhi, Dheeraj Khurana, Roshan Verma

**Affiliations:** 1 Department of Community Medicine & School of Public Health, PGIMER, Chandigarh, India; 2 Department of Pulmonary Medicine, PGIMER, Chandigarh, India; 3 Department of Psychiatry, PGIMER, Chandigarh, India; 4 Department of Cardiology, PGIMER, Chandigarh, India; 5 Department of Pharmacology, PGIMER, Chandigarh, India; 6 Department of Neurology, PGIMER, Chandigarh, India; 7 Department of Otolaryngology, PGIMER, Chandigarh, India

**Keywords:** Behavioral therapy, Tobacco cessation, Tertiary care setting, Randomized controlled trial

## Abstract

**Background:**

To reduce the global burden of tobacco use, clinical guidelines support behavioral therapy and pharmacotherapy as preferred interventions for tobacco cessation. The evidence-based behavioral interventions has consistently shown to be impactful in community settings; however, its efficacy has not been established in hospital settings. The current study aims to investigate impact of trans-theoretical-based behavioral intervention package on tobacco users suffering from non-communicable diseases attending tertiary care settings of North India.

**Methods/design:**

A two-arm randomized controlled trial (RCT) in a tertiary healthcare hospital will be performed. A total of 360 tobacco users attending NCD clinics in four departments, cardiology, neurology, pulmonary medicine, and ENT (otolaryngology), will be recruited over a period of 3 months. After ascertaining the eligibility criteria, they will be followed up to 6 months (1, 3, 6) for their tobacco use status, readiness to quit, nicotine dependence, stage of behavior change, and self-reported and biochemical validation (urine cotinine) for tobacco abstinence. Assignment of intervention including allocation concealment, sequence generation, and blinding will be done as per SPIRIT guidelines for RCT protocols.

**Discussion:**

As no strong evidence exists about the effectiveness of tobacco cessation intervention in tertiary settings, the current study will build evidence about the similar interventions in such settings.

**Trial registration:**

CTRI/2019/09/021406.

## Background

Despite laudable actions against tobacco use for over 50 years, around 1.3 billion people still use tobacco [1], causing more than 8 million deaths each year globally [2]. India alone shares the burden of 267 million tobacco consumers [3], causing 1.2 million people deaths from smoking and exposure to secondhand smoke (SHS) [4]. Furthermore, 2,30,000 deaths result from the use of smokeless tobacco each year [5]. The health consequences of severe tobacco addiction and increased tobacco consumption are tremendous [6, 7]. In particular, the rise of non-communicable diseases (NCDs), including heart disease, stroke, cancer, diabetes, and chronic lung disease, is driven primarily by tobacco use. Findings [8–10] suggests that even quitting tobacco after NCD diagnosis improves survival and quality of life.

In order to combat the problem and reduce the tobacco burden, various targets have been established globally with the initiatives such as WHO Tobacco Free Initiative (1990), WHO framework convention on tobacco control (2003) [11], WHO MPOWER policy (2008) [12], and United Nation’s Sustainable Development Goals (SDG) (2015) [13]. Similarly, the Government of India enacted the Cigarettes and Other Tobacco Products Act (COTPA) [14] in 2003 to prohibit the advertisement of and regulate trade, commerce, production, supply, and distribution of cigarettes and other tobacco products. National Tobacco Control Program (NTCP) was then launched in the year 2008 to ensure effective implementation of COTPA [15].

With the implementation of policies transforming the healthcare system, promoting evidence-based treatment for tobacco cessation is equally important to better address tobacco use and dependence. Treatments including behavioral and pharmacotherapy have shown to be effective when used alone or in combination [16, 17]. Studies have documented that the healthcare providers advice has been successful in 66% increase in quit rate than no intervention [18], and motivational interviewing was more effective than simply providing brief advice for tobacco cessation [19, 20]. For the delivery of the different behavioral interventions, various modalities like web, mobile, telephone, posters, self-help materials, videos, and other sources have gained popularity in the last few decades [21].

Hospitalization increases perceived vulnerability to the harms of tobacco use, thereby motivating the tobacco users to quit. In-person delivery of various modalities especially in hospital settings also encourages patients to quit [22]. Furthermore, after discharge, patients are likely to continue with quitting behavior with positive reinforcement through regular follow-ups [22]. Contrary, multiple challenges account for tobacco dependence treatment in hospital settings. Many clinicians do not consistently offer cessation services to patients due to lack of time due to increase workload, absence of interest, low awareness about treatment, and referral services [23] and lack of resources (drug, counselors) [24]. Secondly, physical structure of the tertiary hospital site creates barriers to implementation, such as lack of private space for intervention requiring sensitive discussion [25, 26]. Furthermore, implementation involving IT innovation also face barriers related to hospital inability to accommodate new systems and staff reporting fatigue towards new initiatives [27].

While there is some evidence on the clinical and cost-effectiveness of behavioral change interventions for quitting tobacco [28–31], there is a lack of data on the effectiveness of these interventions in the tertiary healthcare setting. To our knowledge, merely two randomized controlled trials initiated in hospital settings comparing intensive with brief intervention have been documented so far on tobacco cessation [32, 33], while no such study has been conducted in India. Moreover, earlier research investigated the self-reported change in behavior rather than biochemically verified quit rate using urinary cotinine or other parameters. Therefore, current study plans to establish the evidence for behavioral intervention when delivered among tobacco users in tertiary care hospital setting.

### Objective

To ascertain the differential effectiveness of trans-theoretical model-based behavior change communication strategies for tobacco cessation among tobacco users enrolled from tertiary healthcare settings in North India.

For the current study, we hypothesize that compared with tobacco users in the brief intervention, those in the intensive group will have higher rates of 7-day point prevalent urinary cotinine verified abstinence measured at the 6-month final visit (alternate hypothesis).

## Methods/design

### Study design and setting

The study will be a two-armed randomized controlled trial (RCT) conducted in a tertiary care institute of North India.

The tertiary care health center is at the top level of the healthcare delivery system for the public, which provides specialized consultation care, usually on referral from primary and secondary medical care institutes. They include medical colleges and advanced medical research institutes owned and controlled by the central or state government. The study institute is India's premier medical and research institute, with state-of-the-art educational, medical research, and training facilities. It has bed strength of 1960 across various departments. Four departments, viz. cardiology, pulmonary medicine, neurology, and ENT (otolaryngology), were purposively selected for the study as we expect maximum enrolment of NCD patients in these departments, which is required to assess the effectiveness of the intervention. Apart from the OPD and inpatient services, these departments provide 24 h emergency services and round-the-clock consultation services. The participants visiting four departments, viz. cardiology, neurology, pulmonary medicine, and otolaryngology for their routine check-up will be screened for their eligibility in the study until the target sample is achieved. We expect to have adequate enrolment because the selected institute is a tertiary care institute catering to almost 75,000 participants per day. Moreover, to ensure enrolment in different periods of time, pamphlets will be pasted in outpatient departments and concerned doctors shall be sensitized regarding the study.

### Study participants

While there are no generally agreed-upon definitions of heavy tobacco user, intake of 20 cigarettes per day over 10–20 years, corresponding to 10–20 pack-years, has been associated with a clinically relevant increase in morbidity and used in various studies [6, 7, 34]. Thus, considering the importance of quantifying tobacco exposure and its correlation to the risk of disease, the criteria of at-least 10 pack-years for inclusion of participants, which establishes a baseline smoking or related tobacco products exposure [35], will be used for recruiting the participants visiting tertiary healthcare setting for study. Follow-up period of 6 months was included as we expect a 20% relapse before 6 months follow-up duration [36]. The participants complying with the eligibility criteria will be informed about the study.

### Inclusion, exclusion, and dropout/withdrawal criteria

Participants will be eligible provided they (1) are above 18 years of age, (2) have a history of using tobacco for the last 1 month and at least 10 pack-year or equivalent tobacco, (3) can read and understand English, Hindi, or Punjabi, (4) have a mobile phone with text messages/WhatsApp messages accessibility, and (5) are willing to quit and provide written consent for intervention and follow-up of 6 months in the study.

Participants will be excluded if they (1) are unable to understand any of the languages as mentioned above, (2) are severely ill to participate in the study and require urgent attention for any of the medical problems, (3) are already taking treatment for tobacco cessation, and (4) are mentally ill to accord informed consent for the study.

Participants will be discontinued for the study if they (1) voluntarily withdraw the consent, (2) are not complying with the study schedule, and (3) are not in a condition to continue with the study due to migration or other reasons. The reason for withdrawal will be collected, and no more follow-up will be initiated. Participants who drop out of the study will be considered tobacco users.

Intention-to-treat will be used for data analysis, wherein all participants who are randomized to a treatment arm and receive their assigned intervention are included in the final analysis whether they complete the study or respond to follow-up surveys at study end points. This method will be used to avoid any bias that can potentially arise because of crossover and dropouts, affecting the initial random assignment to treatment groups.

### Sample size

Based on existing literature [37], with a success rate in brief (p1) and intensive (p1) intervention arm of 16.8% and (p2) 30.3% respectively, we expected a difference of approximately 13.5% in cessation rates between the two groups. Sample size was calculated using the Sealed Envelope Ltd. 2012 software application. Power calculator for binary outcome superiority trial based on the formula *n* = *f*(α/2, *β*) × [*p*1 × (100 − *p*1) + *p*2 × (100 − *p*2)]/(*p*2 − *p*1)2 and *f*(*α*, *β*) = [*Φ*-1(*α*) + *Φ*-1(*β*)]2, where *Φ*-1 is the cumulative distribution function of a standardized normal deviate. The alpha level (*α*) at 0.05 and power (*β*) 80% was set to detect a clinically significant difference between the two arms. Sample size was inflated using 20% dropout rate making a total sample size of 366. Participants will be randomly assigned into two arms at a ratio of 1:1 with 183 in each arm. The planned sample size will be recruited in approximately 3 months and followed up for 6 months.

### Assignment of intervention

The PhD scholar (primary author of study) from the Department of Community Medicine and School of Public Health will administer the intervention. She is a dental graduate and has Masters in Public Health (MPH) degree with necessary skills of community intervention and counseling during her training. Also, she witnessed and attended training sessions delivered by counselors and psychiatrist in Drug De-Addiction Treatment Centre of the institute for a period of 3 months, prior to the delivery of intervention.

#### Sequence generation

The participants meeting the inclusion criteria will be randomly assigned by the researcher to either control or intervention arm with a 1:1 allocation as per the randomization sequence generated in advance by a computer program. To ensure a balanced representation of the participants in two groups from various departments, stratified block randomization will be used.

#### Allocation concealment

To ensure and prevent participants and researcher from knowing the study group to which the next participant will be assigned, concealment of allocation will be maintained using sequentially numbered, opaque sealed envelopes. One of the study members other than the researcher will create the allocation sequence in the opaque envelopes for assigning the intervention group, which the researcher will then open for participants’ enrolment.

#### Implementation

Prior to opening envelopes, the eligibility of participants shall be assessed by the researcher. Eligible participants will then be informed about the trial and asked for their participation. After that, the interested participants will then be enrolled in the intervention arms and provided with the assigned intervention by the study researcher.

#### Blinding

**Fig. 1:**
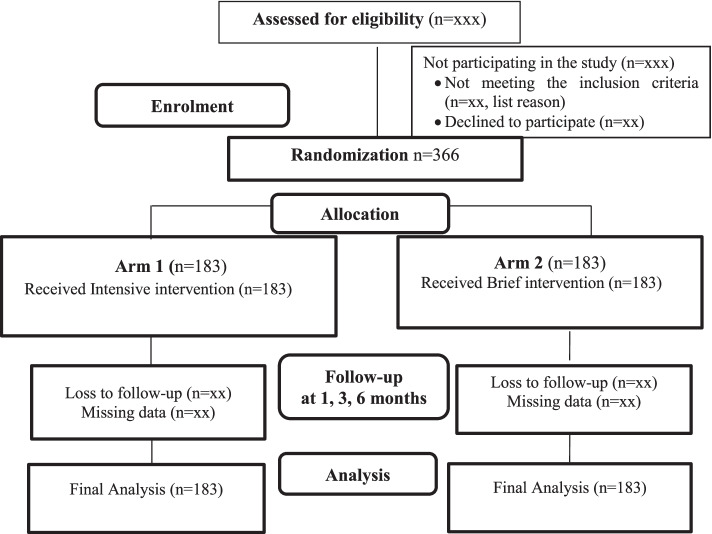
Consort flow chart for enrolment and follow-up plan for randomized controlled trial

The study will be open-label, where participants assigned to the intervention arm will not be blinded, in addition the person delivering the intervention and assessing the outcome (researcher) will be aware of the intervention group to which participants have been assigned (Fig. 1).

### Intervention package

**Fig. 2:**
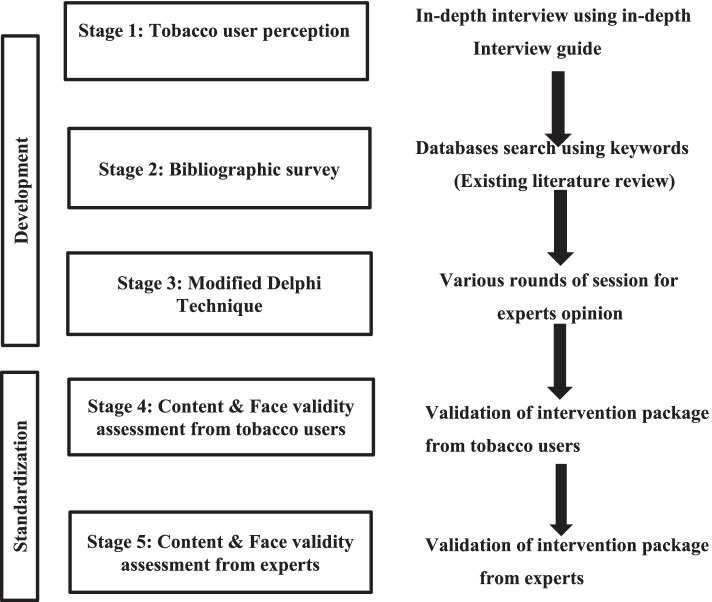
Steps of development and validation of intervention package

The intervention development and evaluation followed the Medical Research Council (MRC) guidelines. The key element of development and evaluation process as per guidelines, i.e., (1) development, (2) feasibility/piloting, (3) implementation, and (4) evaluation, not following a linear sequence, was followed for the current study. Thereafter, the intervention package development, validation, and feasibility assessment was tested before implementing and evaluating its effectiveness in the current study (Fig. 2).

#### Intervention/treatment arm

- *✓ Arm 1: Brief intervention* Face to face counseling, motivational videos, and information leaflet will be included.
- *✓ Arm 2: Intensive intervention* A brief intervention intended to enhance self-efficacy and motivation for quitting will be supported with other modalities in the intensive intervention arm. The supportive modalities including text messages and telephone counseling will enforce the chances of maintaining abstinence. Besides, information to support family and friends during quitting process, tips including coping with cravings, avoiding triggers, and distracting one’s mind from tobacco use will also be included.

The details of the intervention provided in arm 1 (brief intervention) and arm 2 (intensive intervention) are given in Table 1.

**Table 1:** Details of behavior change intervention package in two arms (brief intervention arm and intensive intervention arm) of the study

***Intervention arm***	***Mode and content of delivery***	***Time duration/frequency/provider***	***Benefits***
**Brief and intensive**	(Individual counseling) This will involve face-to-face appointments of patient with researcher aimed to motivate, guide, and psychologically assist tobacco users in quitting. This patient-centered approach enhances an individual’s motivation for change through self-examination and identification of ambivalence to change and the subsequent resolution leading to sustained positive behavior change. It will include asking patients about their tobacco use status, and discuss the options that exist to support a quit attempt	10–20 min/once during first contact/researcher	Both minimal (< 20 min in 1 visit) and intensive (≥ 20 min plus > 1 follow-up visit) physician-advice interventions effectively increase the proportion of adults who successfully quit tobacco and remain abstinent for ≥ 6 months [8]. There is a dose–response relationship between the intensity of counseling and cessation rates (i.e., more or longer sessions improve cessation rates) [38]
	(Motivational videos) Videos based on real life stories of patients with tobacco use history will educate and sensitize patients and care givers on harmful effects of tobacco and motivate patients to quit	3–5 min/once during first contact/researcher	Technology including mobile phones, the internet, and social media platforms, can increase access to care by extending the work of counselors and overcoming the geographical barriers. These supports can increase the likelihood of adults quitting compared with no intervention and can be a cost-effective adjunct to other treatments [39]
	(Patient information leaflet (PIL)) Patient information leaflet will be based on specific characteristics or concerns of tobacco users and include written material aiming to educate patients about the disease, ill effects of using tobacco on health, benefits of quitting and how to resist cravings and avoid relapse	Once at first contact/researcher	Providing self-help materials (primarily print-based) tailored to the individual patient (that is, beyond a brochure that simply describes the health effects of tobacco use and benefits of cessation) is effective in improving tobacco abstinence [40]
**Intensive**	(Telephone counseling) Counseling through regular telephone follow-up will provide support and encouragement to individuals who uses tobacco and want to quit or individuals who have recently quit	05–15 min/five times at < 1, 4, 6, 9, and 12 weeks in 3 months after enrolment/researcher	Telephone-based access to counseling and smoking cessation resources increases cessation rates. The majority of helplines provide access to individual counseling; the greatest amount of counselor contact, the greater the likelihood of successful cessation [41]
	(Text messages using SMS) Messages will include short text based on the current stage of behavior as per trans-theoretical model for their motivation and support regarding tobacco cessation and maintaining quit status	Bi-weekly for 3 months/researcher	Technology, including mobile phones, the internet, and social media platforms, has the power to increase access to care by extending the work of counselors and overcoming geographical barriers. These supports can increase the likelihood of adults quitting compared with no intervention and can be a cost-effective adjunct to other treatments [39]

The intervention to be delivered was adapted by collecting data from stakeholders, including health professionals, and interviewing tobacco users from the setting.

### Data collection

After fulfilling the inclusion criteria and seeking written consent, the researcher will collect data from tobacco users enrolled in the study. Baseline and end of follow-up (6 months) of intervention assessment will include assessment of tobacco use (daily tobacco consumption, age of initiation, quit attempt, treatment sought for cessation (if any)); knowledge, attitude, and practices about tobacco use.

The assessment of motivation to quit tobacco will be undertaken using Readiness to Change questionnaire [42] (RCQ), nicotine dependence using FTND scale [43], and stages of behavior change using a trans-theoretical model of behavior change at each follow-up (0, 1, 3, 6) months. The self-reporting assessment for 7-day point prevalence and continuous tobacco abstinence will also be done at each (0, 1, 3, 6) follow-up month. In addition, a urine sample will be collected at the last follow-up (6 months of intervention) to confirm the cotinine presence biochemically (Table 2).

**Table 2:**
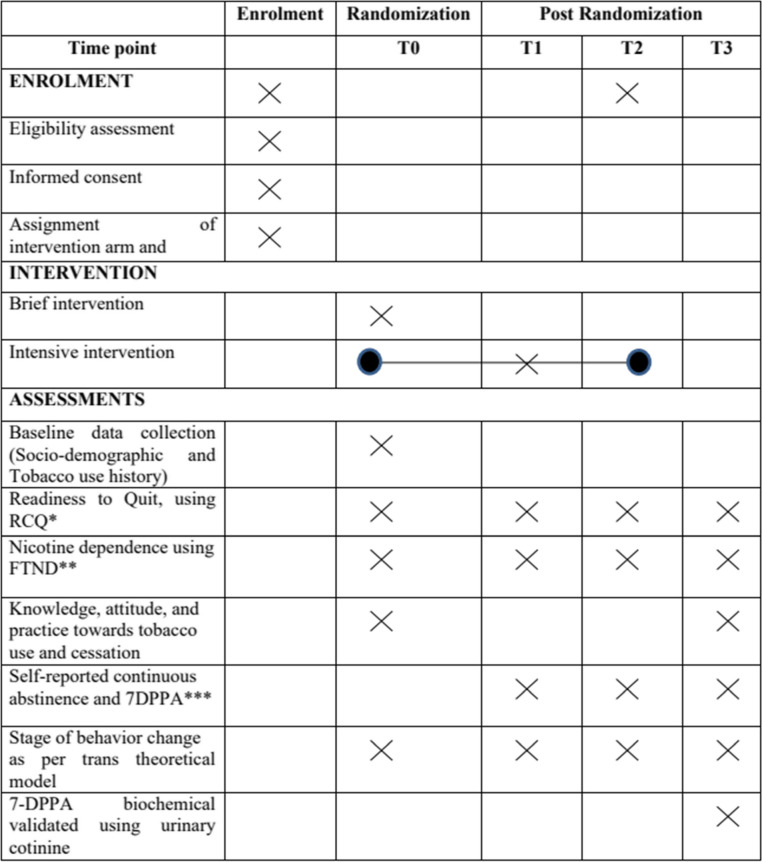
Data collection items and schedule at baseline and follow-up in the study

^*^Readiness to change questionnaire

^**^FTND Fagerstrom Tobacco Nicotine Dependence

^***^7DPPA Seven-day point prevalence abstinence

T0: baseline, T1: 1 month follow-up, T2: 3 months follow-up, T3: 6 months follow-up

Sample handling: Urine sample will be collected in container with a lid to prevent leakage and then transported from the collection to storage site using dry ice in an ice box. At the storage site, the collected sample will be stored in refrigerator maintained at − 80° temperature with back-up generator system to provide power during an electrical outage. Proper labeling to withstand the storage condition, i.e., good label material will be used to have readable printing even after long-term storage. After performing testing procedure and analysis, the samples will be discarded after mixing with sodium hypochlorite solution. Biological waste management guidelines followed in the institute (PGIMER Chandigarh) and biological sample collection, processing, storage, and information management details provided in the reference will be followed for sample handling during the study [44].

### Outcome measures

#### Primary outcome

- Validated 7-day point prevalence tobacco abstinence at 6 months (last follow-up) measurement using urinary cotinine

#### Secondary outcomes

Measures at each follow-up period 1, 3, and 6 months:

- Self-reported continuous tobacco abstinence
- Self-reported 7-day point prevalence tobacco abstinence
- Self-reported tobacco use reduction
- Readiness to change by RCQ
- Scores of Nicotine dependence by FTND instrument
- Number of quit attempts and relapses at each follow-up

Measures at follow-up 6 months:

- Change in knowledge, attitude, and practices score at 6 months (Table 3)

**Table 3:** Study outcome measures at different time intervals

**Outcomes**	**Domain**	**Specific measurement**	**Specific metric**	**Method of aggregation**	**Time points**
*Validated 7-day PPA*	Abstinence status	Cotinine (biochemically)	Abstinence	Proportion	6 months
*Self-reported CA*	Abstinence status	Self-reported	Abstinence	Proportion	1, 3, 6 months
*Self-reported 7DPPA*	Abstinence status	Self-reported	Abstinence	Proportion	1, 3, 6 months
*Knowledge, attitude, and practices (KAP)*	Knowledge, attitude, and practices	Questionnaire	Change from baseline	Mean	6 months
*Nicotine dependence*	Dependence score	FTND	Change from baseline	Mean	1, 3, 6 months
*Quit attempts*	Quit attempts	Follow-up questionnaire	Presence of quit attempts	Number of quit attempts	1,3, 6 months
*Readiness to change*	Stage of behavior change	Readiness to change questionnaire	Change in stage from baseline	Proportion	1,3, 6 months
*Reduction in tobacco use*	Reduction tobacco use	Quantity	Change from baseline	Proportion	1, 3, 6 months

### Data analysis

Data analysis will be undertaken by the investigators using the SPSS statistical analysis package.

Analysis of primary outcome of 7-day point prevalence tobacco abstinence at 6 months will be based on an intention-to-treat approach. It shall be done by estimating the mean difference in percentage of quit rates between the intensive and brief intervention groups at 6 months.

Regression analysis and /or ANOVA will be used for primary and secondary outcomes. For not normally distributed data, robust standard errors, truncation, or transformation will be used. For missing data, multiple imputations will be used. The net changes in primary and secondary outcome measures will be considered by exploratory analysis. Marginal means and treatment effect with its associated 95% CI and probability values will be presented and reported. The conventional significance level of 0.05 will be used in all analyses to reject the null hypothesis of no difference between two groups.

## Discussion

To explain behavior change, many behavioral change theories, like the health belief model [45], socio-cognitive theory [46], and trans-theoretical framework [47], are being used, focusing upon different factors. The current study will consider the trans-theoretical framework to address the change in behavior towards tobacco cessation after providing an intervention. This framework is used for following reasons: firstly, this helps to assess an individual's readiness to act on a new healthier behavior. Secondly, it provides strategies or processes of change to guide the individual. Third, this framework will emphasize the importance of tobacco users' motivation and self-efficacy while considering the barriers to change and cues to action. Studies worldwide using trans-theoretical model (TTM) to track tobacco use behavior have established its validity and reliability across various settings [48–52]. Furthermore, this framework will help clinicians track tobacco users' movement from one stage to the next stage [53].

The tobacco cessation intervention (TCI) has variably shown the effectiveness and efficacy of reducing ill health and increasing quality-adjusted life years (QALY). A systematic review [54] concluded the cost-effectiveness of intensive over brief tobacco cessation intervention with 960 and 280 discounted cumulative number of QALYs per year respectively gained in the two interventions. Further studies documented intensive tobacco cessation intervention in reducing ill-health, morbidity, and mortality compared to brief intervention [55]. As no evidence could be reported on the comparative effectiveness of intensive over the brief intervention for tobacco cessation in the Indian hospital setting, the results of current study will provide quality evidence to replicate the protocol in similar settings across the globe.

Despite the strong evidence about the effectiveness of tobacco cessation intervention in community settings, its implementation by health professionals in tertiary care has still not been explored. Tobacco-related diseases are one of the main reasons for all general hospital admissions [56]. During hospital stays, these patients should be advised to stop tobacco use because this decision has been related to reductions in morbidity and mortality [57]. Although many healthcare delivery centers restrict or prohibit patients from tobacco use to protect other patients and staff from the effects of passive smoking, this tobacco-free environment may provide an opportunity for hospitalized patients to attempt abstaining from its use.

Nevertheless, the use of intervention for cessation has been frequently ignored by health professionals in hospitalized populations and often discharge patients from tertiary care without sufficiently addressing opportunities for tobacco prevention [58, 59]. For this reason, providing (or at least initiating) tobacco dependence treatments in hospitals may be an effective preventive health strategy [60, 61]. To our knowledge, no study in an Indian setting has investigated the effects of tobacco-cessation intervention in a subset of patients admitted in a tertiary healthcare delivery center. Thus, the study will build evidence on the effectiveness of interventions in such settings and advocate for a tailored intervention.

Most of the existing studies relied on questionnaire methods such as Russell Standard for self-reporting abstinence rather than biochemical verification to measure tobacco use status. The biochemical verification shall increase rigor and validity compared to self-reported tobacco abstinence. However, it also has limitations, including the inability to confirm long-term abstinence, implementation challenges, and high performance cost.

The study has several strengths and endeavors to strengthen the theoretical framework for tobacco cessation interventions. Firstly, this will be the first comprehensive study from India conducted in tertiary care setting comparing intensive with brief intervention for tobacco cessation. Secondly, it will use a holistically designed intervention developed after obtaining all stakeholders’ views, including tobacco users. Thirdly, it shall examine the effect of tobacco cessation intervention after 1, 3, and 6 months of providing intervention, which will provide an opportunity to evaluate long-term treatment effects on tobacco abstinence. At last, verification of quitting status with cotinine assessment will strengthen the validation of outcomes. There are few possible limitations of the study. First, the possibility of missing data throughout the follow-up period cannot be ignored, which shall influence the validity and internal reliability of the results. However, this is a frequent phenomenon of any long-term trial involving tobacco cessation. Second, the results may not be generalized for the general population as the participants will be from the hospital settings in the study. Moreover, the effect of secondhand smoke (SHS) in the measurement could not be ascertained. Social context in tobacco has been widely cited as integral to understanding why, how, where, and with whom people use tobacco, along with and the non-random social distribution of tobacco use. Although the need to incorporate the social context has now been recognized by many of the disciplines involved in tobacco control research, its measurement was out of scope of the study.

The current study has few policy and programmatic implications. As the intervention package for tobacco cessation has been developed through a formative research, evidence shall inform and provide insights on its acceptability and feasibility at a larger scale across several centers and countries. With limited use of health system resources such as healthcare personnel (counselors) and space, the findings of this study will provide direction to policy makers, implementers, and educators for implementing the intervention in their settings. Since research in this area is in its infancy in LMIC and MICs, therefore, this will provide an impetus for researchers working on tobacco cessation to generate new evidence in real-time practice. 

### Dissemination of study results

The evidence generated on the effectiveness of tobacco cessation intervention in India’s tertiary care setting will be communicated at scientific meetings and submitted for publication in peer-reviewed journals for more comprehensive readability. Besides, package developed and evaluated from current study will provide evidence for large-scale future research and will provide opportunity to motivate tobacco using patients with better access to behavioral change intervention such as videos, information material, and messages for tobacco cessation. ClinicalTrials.gov record will also be updated regularly.

## Trial status

Recruitment of participants started in December 2020 and is currently ongoing. The study findings are expected to be available in August 2022.
